# Correction: Vocal tract allometry in a mammalian vocal learner

**DOI:** 10.1242/jeb.245305

**Published:** 2022-12-16

**Authors:** Koen de Reus, Daryll Carlson, Alice Lowry, Stephanie Gross, Maxime Garcia, Ana Rubio-Garcia, Anna Salazar-Casals, Andrea Ravignani

There was an error in *J. Exp. Biol.* (2022) **225**, jeb243766 (doi:10.1242/jeb.243766).

The ORCID iD for Alice Lowry should read: 0000-0003-2902-7568.

Additionally, in the first sentence of Materials and Methods, the number of harbour seals is incorrectly given as ‘35 males, 33 females’; it should instead be ‘34 males, 34 females’. The number of males and females is correctly reported in paragraph two of Materials and Methods, Table S1 and the dataset, and does not affect the results in the article or the conclusions of this study.

Both the online full-text and PDF versions of the paper have been corrected. The authors apologise for these errors and any inconvenience they may have caused.

