# Supplementary material for: Vocal tract allometry in a mammalian vocal learner
Source: J Exp Biol. 2022 Apr 28;225(8):jeb243766. doi: 10.1242/jeb.243766 (PMC9124484; doi:10.1242/jeb.243766)
Supplement: Supplementary information [file jexbio-225-243766-s1.pdf]

Supplementary Materials and Methods

Acoustic analyses

We ran several acoustic analyses to show that seals can escape acoustic allometry. The acoustic data used to perform these analyses comes from the study published by Torres Borda and colleagues (2021), during which they observed fundamental frequency changes in harbour seal pups under different noise conditions (silence, low and high noise). The acoustic data was complemented with previously unpublished body mass measurements of the animals in their study. The harbour seal pups were weighed on their day of arrival at the Sealcentre Pieterburen, a pinniped rehabilitation centre in the Netherlands, where the animals were also audio recorded. Eight harbour seals participated in the noise playback experiment and the fundamental frequency (f_0) was extracted from the vocalisations they produced during the testing period (all details in Torres Borda et al., 2021).

Grouping the observations by seal ID and noise condition, we computed the median f_0 for each of the 24 groups. We then regressed median f_0 on body size (using body mass as a proxy for body size) for each of the noise conditions (Figure 2A). Visually, an inverse relationship between body size and call frequency seems to hold in all three noise conditions, but none of the correlations ($\tau_{silence}$ = -0.18, τ_{low} = -0.25, τ_{high} = -0.40) are significant ($p < 0.05$). This apparent inconsistency may be explained by a large degree of overlap in the range of f_0 values produced by individuals of differing body size between noise conditions. For instance, an animal of 12.4 kg under silence can produce a similar f_0 value as an animal of 7.3 kg under high noise (see Figure 2A), suggesting that acoustic allometry may not hold across noise conditions. Could it be that the environmental noise conditions in which vocalisations are produced more strongly affect the f_0 values than body size? If hypothetically we were to record calls of harbour seal pups on different days and irrespective of environmental noise conditions, the inverse relationship between f_0 and body size may disappear (i.e., acoustic allometry would break) if the individuals can, thanks to their large vocal plasticity, adjust their f_0 depending on the noise conditions.

To assess if allometric relationships do indeed break down across noise conditions, we computed 10,000 different combinations of randomly selected median f_0 values (1 of the 3 median frequency values per seal) and matched each value to the corresponding body mass value. We then performed 10,000 Kendall rank correlations, each among the 8 resulting pairs of f_0 and body mass values. Figure

Journal of Experimental Biology • Supplementary information

2B shows the kernel density distribution of the resulting correlation coefficients and their associated p-values (Figure 2C). We find that the median correlation coefficient is -0.18, suggesting a weak negative correlation. The median p-value is 0.38, indicating that—in more than half of the cases—we cannot reject the null hypothesis which states that the correlations are generally not significantly different from 0. In only 2.2% of cases (217 out of 10,000) is the correlation significant. We should take care when interpreting the correlation p-values as the power of the test statistic is low given the small sample size (n = 8), resulting in a higher probability of committing type II errors. Moreover, the body mass values correspond to the measurements taken on the day of the animal's arrival at the Sealcentre; they are not representative of the actual body mass values on the days of testing. Using the same set of random combinations of f_0 values, we also plotted the density distribution for the linear regression coefficients (Figure 2D). The median regression coefficient is -10.8 Hz/kg. The difference in initial body mass between the largest and smallest seal is 5.1 kg. This means that across their mass range, we would expect, on average, a 55.08 Hz difference. For every seal, we calculated the range between the median f_0 values of the silent and high noise condition (silence f_0 – high f_0) and find that the median is 73.6 Hz. This suggests that the differences caused by individual variability in f_0 in response to noise conditions are larger than the f_0 differences expected from body mass differences alone. Seals of differing body sizes (e.g., 7 vs. 12 kg) could thus potentially produce the same f_0 value (and they actually do, see Figure 2A). Furthermore, we also calculated, for each seal, the f_0 range (maximum – minimum f_0) for all recorded observations from that individual. We find that, across the tested seals, the median f_0 range is 322.6 Hz. Applying the same logic as above, seals with a body mass difference of almost 30 kg (322.6 / 10.8) could all produce similar f_0 values. Finally, we computed and compared two simple generalised linear models, testing if body mass (Model 1: f_0 ~ Body Mass) or noise condition (Model 2: f_0 ~ Noise Condition) was better at predicting f_0. We find that body mass is not a significant predictor of f_0 (t = -1.78, p = 0.09), but noise condition is ($t_{high\ vs.\ low}$ = 2.10, p = 0.048; $t_{high\ vs.\ silence}$ = 3.90, p = 0.001). Moreover, Model 1 explained 12.63% of the deviance (calculated as (1 - residual deviance / null deviance) * 100) and Model 2 explained 42.05% of the deviance. An ANOVA test confirmed that Model 2 significantly outperformed Model 1 (F = 10.7, p = 0.001), showing that environmental noise conditions may have a stronger influence on f_0 than body size.

Journal of Experimental Biology • Supplementary information

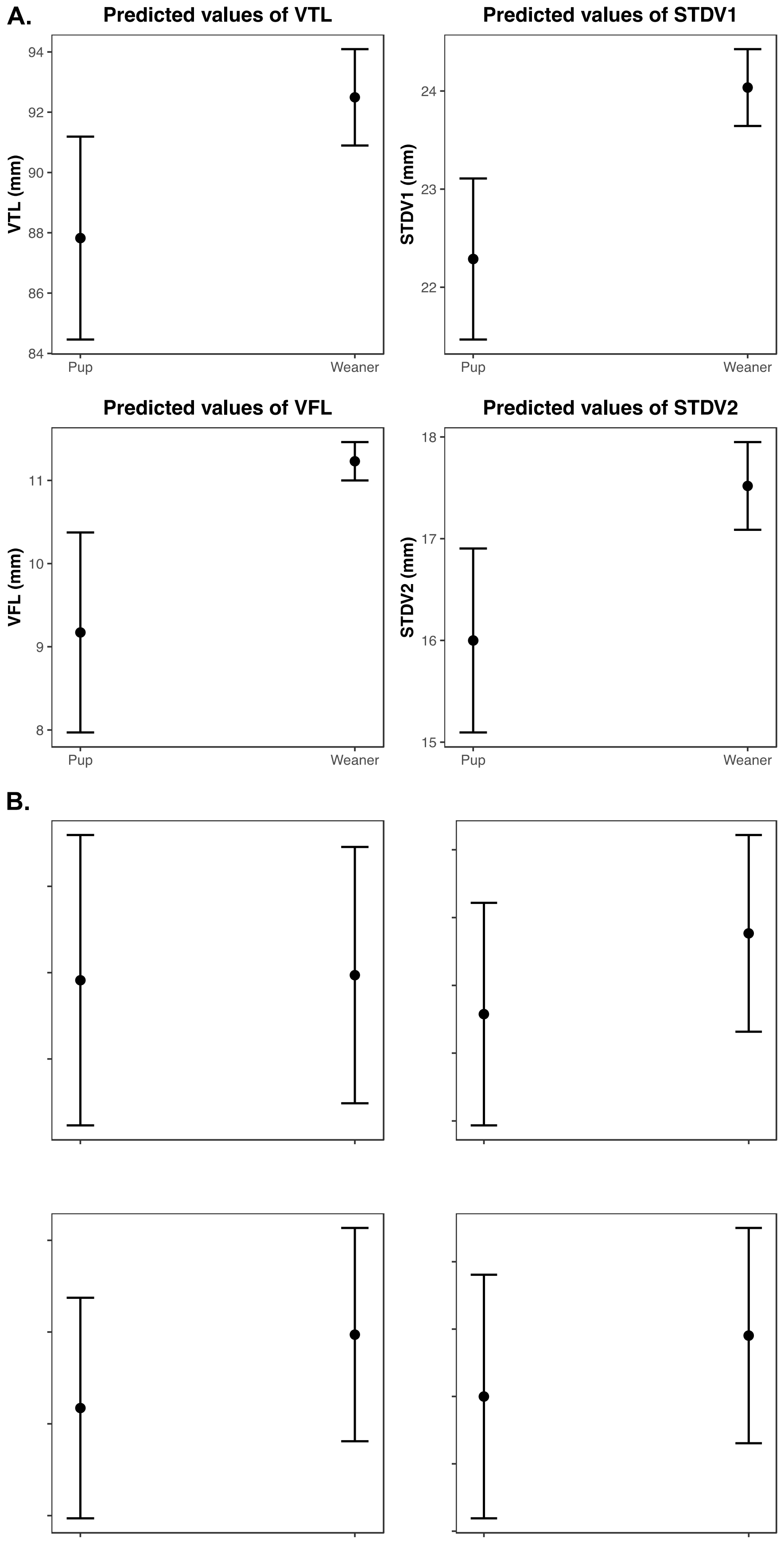

Fig. S1. Predicted effects of A) Age class and B) Sex in each of the GLM models.

Journal of Experimental Biology • Supplementary information

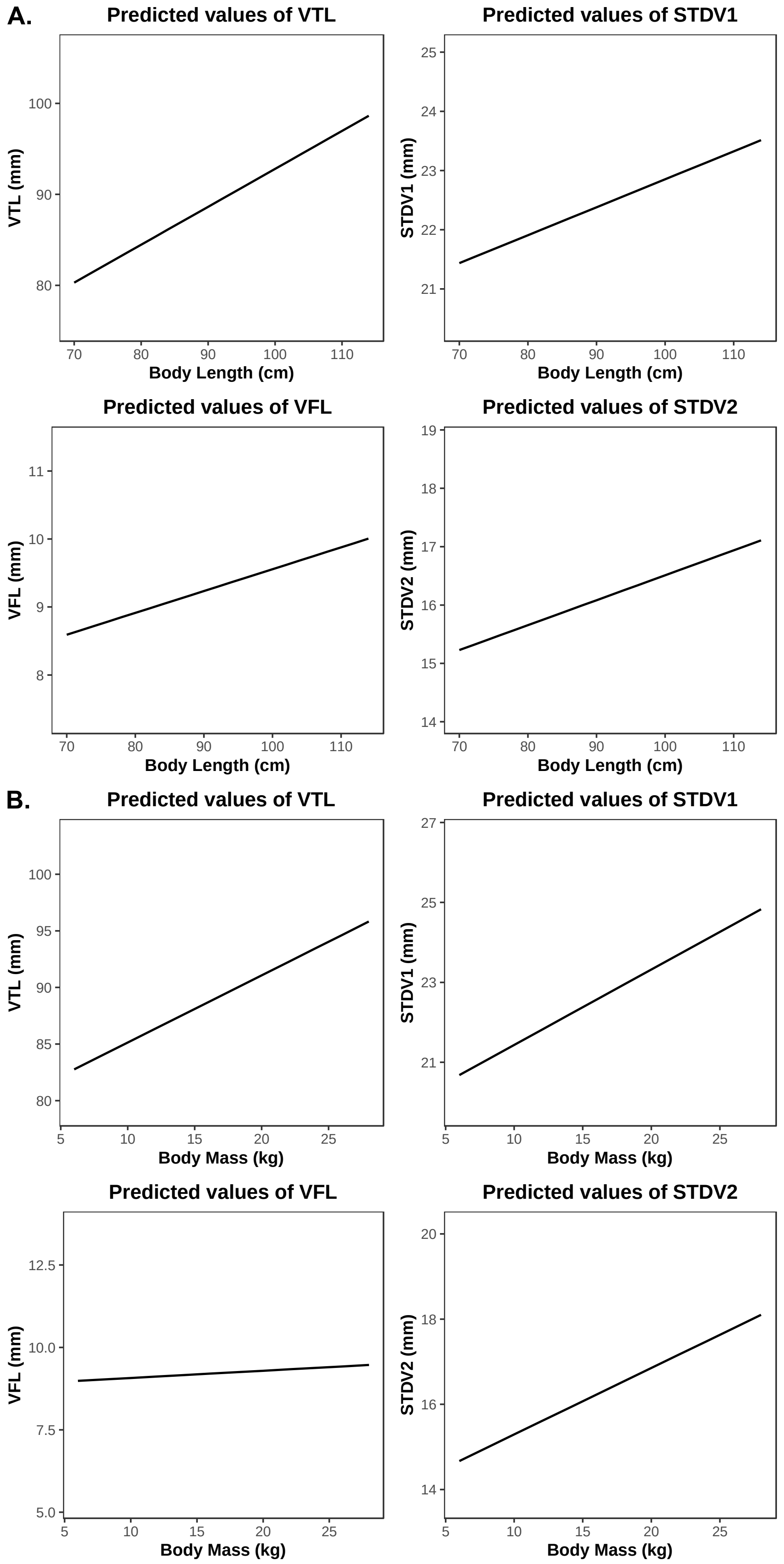

Fig. S2. Predicted effects of A) Body Length and B) Body Mass in each of the GLM models.

Journal of Experimental Biology • Supplementary information

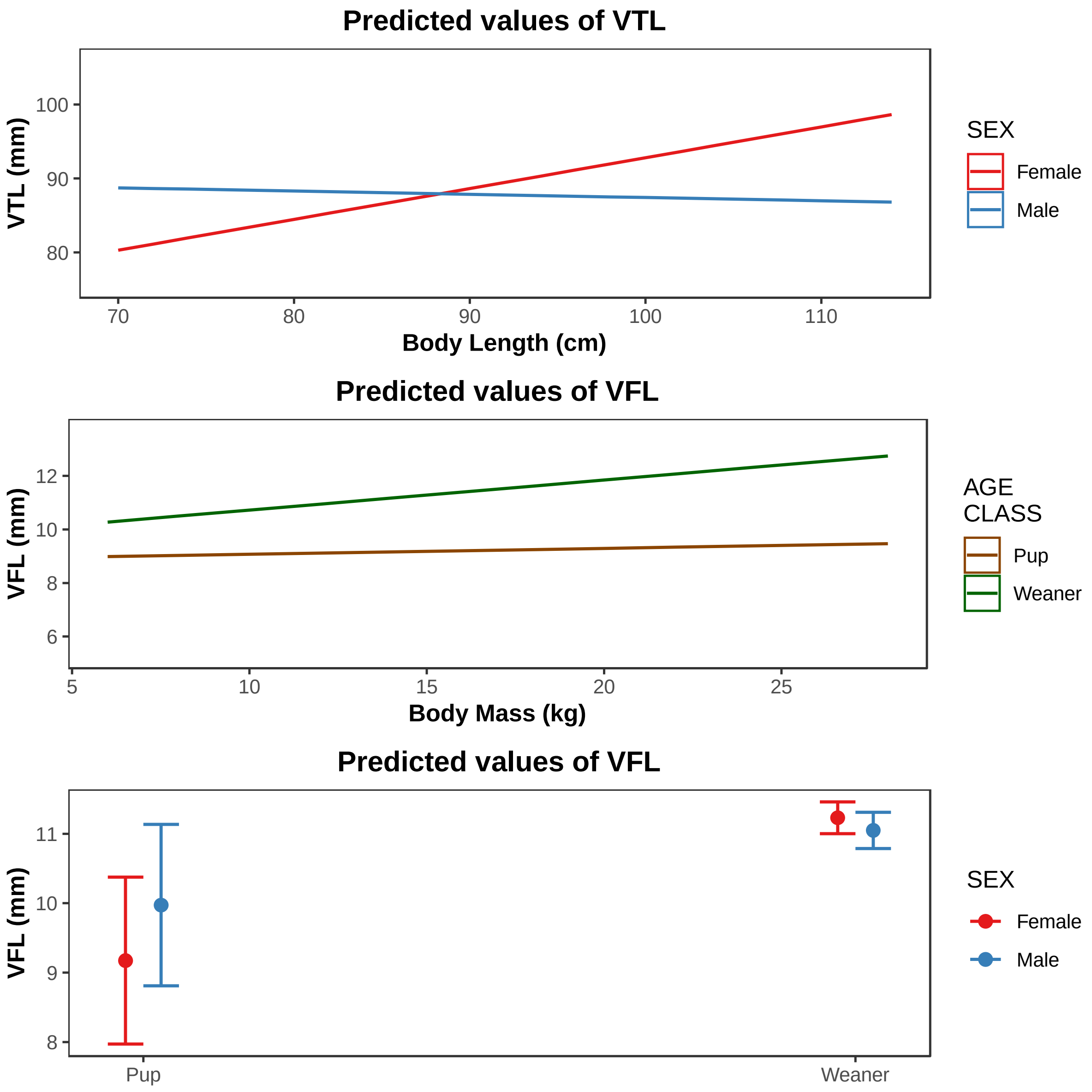

Fig. S3. Predicted effects of the body length and sex interaction for VTL (top), the body mass and age interaction for VFL (middle) and the age and sex interaction for VFL (bottom).

Journal of Experimental Biology • Supplementary information

Table S1. *List of sampled animals*

ID	Age class	Where from	Sex	Body Length (cm)	Body Mass (kg)	Girth (cm)	Cause of death
1	weaner	NL	F	86	15.6	90	Euthanised
2	weaner	NL	F	99	17.3	83	Died during rehab
3	weaner	NL	M	96	26.8	76	Found dead in the wild
4	weaner	NL	M	96	22.9	71	Euthanised
5	weaner	NL	M	86	19.2	71	Died before rehab
6	weaner	NL	M	84	18.2	69	Euthanised
7	weaner	NL	M	89	19.9	66	Euthanised
8	weaner	NL	F	94	14.2	66	Euthanised
9	weaner	NL	F	92	15.8	65	Euthanised
10	weaner	NL	F	94	14.9	65	Died during rehab
11	weaner	NL	M	104	20.8	64	Died before rehab
12	weaner	NL	F	86	16.5	63	Died during rehab
13	weaner	NL	F	86	15.7	63	Euthanised
14	weaner	NL	M	100	18.37	62	Euthanised
15	weaner	NL	F	93	16.8	62	Euthanised
16	weaner	NL	M	114	18.8	61	Died before rehab
17	weaner	NL	F	87	15.8	61	Euthanised
18	weaner	NL	F	93	17.8	60.5	Euthanised
19	weaner	NL	M	96	16.3	60	Died during rehab
20	weaner	NL	F	82	15.3	60	Euthanised
21	weaner	NL	M	80	14.3	60	Died before rehab
22	weaner	NL	F	88	16.1	59	Died during rehab
23	weaner	NL	F	89	16.9	58	Euthanised
24	weaner	NL	F	71	10	58	Euthanised
25	weaner	NL	M	92	17	57	Euthanised
26	weaner	NL	F	94	14.5	57	Died during rehab
27	weaner	NL	M	86	13.9	57	Died before rehab
28	weaner	NL	F	79	11.9	56.5	Euthanised
29	weaner	NL	M	85	14.6	56	Died during rehab
30	weaner	NL	M	94	14.6	55	Euthanised
31	weaner	NL	F	92	13.7	55	Died during rehab
32	weaner	NL	M	80	13.1	55	Died during rehab
33	pup	NL	F	75	11.9	55	Euthanised
34	pup	NL	M	84	11.79	54	Found dead in the wild
35	weaner	NL	F	93	14	53	Died during rehab
36	pup	NL	M	83	11.47	52	Euthanised
37	weaner	NL	F	93	13.9	51.5	Euthanised

Journal of Experimental Biology • Supplementary information

38	weaner	NL	M	86	13	51.5	Died before rehab
39	weaner	NL	F	87	12.4	51	Died before rehab
40	pup	NL	M	86	10.6	51	Euthanised
41	pup	NL	F	81	11.37	49.5	Euthanised
42	pup	NL	F	82	9.3	49	Found dead in the wild
43	pup	NL	M	80	9.46	47	Euthanised
44	pup	NL	M	73	8.6	46	Found dead in the wild
45	pup	NL	F	77	8.5	44.5	Died before rehab
46	pup	NL	F	80	9.63	44	Found dead in the wild
47	weaner	NL	M	87	9.3	44	Died during rehab
48	pup	NL	F	70	8	44	Found dead in the wild
49	pup	NL	M	87	9.43	41	Found dead in the wild
50	weaner	NL	F	77	7.47	40	Euthanised
51	pup	NL	M	80	7.28	38.5	Died during rehab
52	pup	NL	M	80	9.95	36	Died before rehab
53	weaner	DE	M	85.5	19.2	67.5	Mercy killed
54	weaner	DE	F	98.5	17	66	Mercy killed
55	weaner	DE	M	90	14.6	65	Mercy killed
56	weaner	DE	M	101	20.8	64	Mercy killed
57	weaner	DE	M	90	17	63	Found dead in the wild
58	weaner	DE	F	92	20.4	62	Found dead in the wild
59	weaner	DE	F	99	17.8	60.5	Mercy killed
60	weaner	DE	M	86	16	60.5	Found dead in the wild
61	weaner	DE	M	90	16.6	59	Found dead in the wild
62	weaner	DE	F	94	17	58	Found dead in the wild
63	weaner	DE	M	90	14.6	57	Mercy killed
64	weaner	DE	M	96	13.4	56	Found dead in the wild
65	weaner	DE	F	82	11.6	55.5	Found dead in the wild
66	weaner	DE	F	97	14.8	54	Mercy killed
67	weaner	DE	M	81	10.2	51	Mercy killed
68	weaner	DE	F	88.5	12	50	Mercy killed

Note. Seals were from the Netherlands (NL) or Germany (DE). Sex is denoted as F for females and M for males.

Journal of Experimental Biology • Supplementary information

Table S2. *Pairwise Spearman correlations for pups and weaners*

Age class	Variable	Body Length (cm)	Body Mass (kg)	Girth (cm)	VTL (mm)	VFL (mm)	VFT (mm)	STDV1 (mm)
Pups	Body Mass (kg)	0.40						
	Girth (cm)	0.23	0.72					
	VTL (mm)	0.23	-0.03	-0.04				
	VFL (mm)	0.22	0.08	-0.01	0.49			
	VFT (mm)	-0.21	0.11	0.42	0.01	0.16		
	STDV1 (mm)	0.20	0.18	0.08	0.49	0.79*	0.55	
	STDV2 (mm)	0.35	0.36	0.38	0.26	0.71	0.57	0.76*
Weaners	Body Mass (kg)	0.51*						
	Girth (cm)	0.28	0.76*					
	VTL (mm)	0.39*	0.48*	0.39*				
	VFL (mm)	0.58*	0.64*	0.41*	0.54*			
	VFT (mm)	0.16	0.48*	0.50*	0.22	0.34		
	STDV1 (mm)	0.38*	0.61*	0.38*	0.46*	0.67*	0.47*	
	STDV2 (mm)	0.32	0.48*	0.31	0.47*	0.57*	0.30	0.66*

Note. * indicates $p < .05$ after correcting for multiple comparisons using the Holm-Bonferroni method.

Table S3. *Generalised linear model (GLM) estimates for all vocal structures*

Vocal structure	Effect	Estimate	Std.Err.	2.5%	97.5%	p
VTL	Intercept	42.4788	9.2445	23.9898	60.9678	< 0.001
	Age Class-Weaner	4.6695	1.7801	1.1093	8.2297	< 0.05
	Body Length	0.4170	0.1184	0.1802	0.6538	< 0.001
	Body Mass	0.5933	0.2182	0.1569	1.0297	< 0.01
	Sex-Male	40.7192	12.1685	16.3822	65.0562	< 0.01
	Body Length*Sex-Male	-0.4610	0.1379	-0.7368	-0.1852	< 0.01
VFL	Intercept	9.1651	3.0598	3.0455	15.2847	< 0.01
	Age Class-Weaner	-2.7492	3.2348	-9.2188	3.7204	0.399
	Body Length	-0.0189	0.0396	-0.0981	0.0603	0.635
	Body Mass	0.1050	0.0301	0.0448	0.1652	< 0.001
	Sex-Male	1.0070	0.3670	0.2730	1.7410	< 0.01
	Age Class-Weaner*Body Length	0.0562	0.0411	-0.026	0.1384	0.177
	Age Class-Weaner*Sex-Male	-1.1833	0.4071	-1.9975	-0.3691	< 0.01
STDV1	Intercept	15.389	1.6827	12.0236	18.7544	< 0.001
	Age Class-Weaner	1.7474	0.4352	0.8770	2.6178	< 0.001
	Body Length	0.0472	0.0236	0.0000	0.0944	< 0.05
	Body Mass	0.1887	0.0533	0.0821	0.2953	< 0.001
	Sex-Male	0.5956	0.2754	0.0448	1.1464	< 0.05
STDV2	Intercept	9.9726	1.8526	6.2674	13.6778	< 0.001
	Age Class-Weaner	1.5194	0.4792	0.5610	2.4778	< 0.01
	Body Length	0.0427	0.0260	-0.0093	0.0947	0.105
	Body Mass	0.1560	0.0587	0.0386	0.2734	< 0.01
	Sex-Male	0.4523	0.3032	-0.1541	1.0587	0.141

Note. The vocal tract structures tested are vocal tract length (VTL), vocal fold length (VFL), subglottic tracheal dorsoventral distance 1 (STDV1) and subglottic tracheal dorsoventral distance 2 (STDV2). For all models, the reference level for Age Class is ‘Pup’ and the reference level for Sex is ‘Female’.
